# Chronic tophaceous gout presenting as acute arthritis during an acute illness: a case report

**DOI:** 10.1186/1757-1626-1-238

**Published:** 2008-10-15

**Authors:** Abhijeet Dhoble, Vijay Balakrishnan, Robert Smith

**Affiliations:** 1 Department of Internal Medicine, Michigan State University, East Lansing, Michigan, USA

## Abstract

**Background:**

Gout is a metabolic disease that can manifest as acute or chronic arthritis, and deposition of urate crystals in connective tissue and kidneys. It can either manifest as acute arthritis or chronic tophaceous gout.

**Case presentation:**

We present a 39-year-old male patient who developed acute arthritis during his hospital course. Later on, after a careful physical examination, patient was found to have chronic tophaceous gout. The acute episode was successfully treated with colchicines and indomethacin.

**Conclusion:**

Gout usually flares up during an acute illness, and should be considered while evaluating acute mono articular arthritis. Rarely, it can also present with tophi as an initial manifestation.

## Background

Gout is a metabolic disease, which is characterized by acute or chronic arthritis, and deposition of monosodium urate crystals in joint, bones, soft tissues, and kidneys [1-4]. In 18^th ^century, Garrod proposed a causative relationship between elevated uric acid and urate crystal formation, which is underlying pathology for gout [4]. Gout can either manifest as acute arthritis or chronic arthropathy, which is also called tophaceous gout [1,2,5].

## Case presentation

A 39-year-old African American male patient was admitted with one-day history of acute left lower quadrant pain, and was diagnosed with acute uncomplicated diverticulitis, confirmed by computed tomography (CT) of the abdomen. His medical and surgical history was unremarkable, and he denied any medication use. He denied smoking or illicit drug use, but admitted occasional alcohol use on every other weekend. He did not follow any particular diet. He had an average built with BMI of 29.6. He was started on intravenous antibiotics and pain medication, which led to significant clinical improvement within two days.

**Figure 1:**
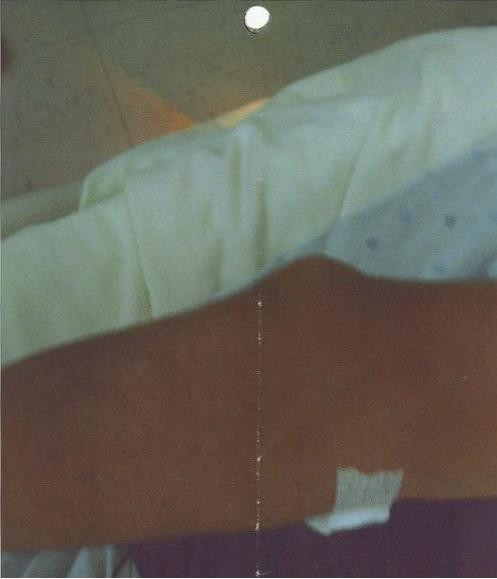
Tophus at the back of right elbow.

**Figure 2:**
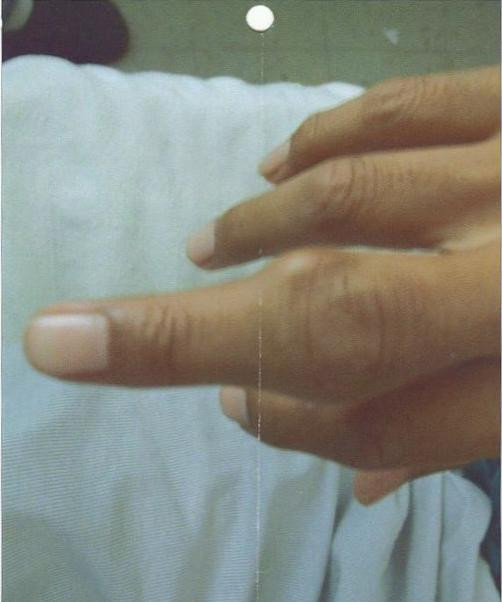
Tophi/tophus around the proximal inter-phalangeal joint of right middle finger.

On the third day of hospitalization, he developed acute, severe pain and swelling of the left elbow. Within next few hours, pain worsened and he was unable to move the elbow joint, which was tender, erythematous, and swollen on examination (figure 1). Never investigated in the past, we also noted a firm 4 × 6 cm mass on each elbow, and another one surrounding the proximal inter-phalangeal joint of right middle finger (figure 2). There was no overlying edema or cellulitis. There were no other swellings or tophi noted especially on toes or ears. When asked particularly, he denied similar episodes in the past. He also denied any episode of swelling of great toe in the past.

Plain radiography of left elbow showed joint effusion, and soft tissue swelling. Radiography of other joints including hands and feet was not performed. Laboratory values on the third day are given in table 1. Liver function test was also performed, and the results were unremarkable. Diagnostic arthocentesis was performed on both the elbows, and revealed negatively birefringent needle-shaped crystals using polarized microscopy in both samples. Detailed analysis of synovial fluid is given in table 2. The swelling on the right elbow was aspirated to determine the etiology because patient had that swelling for a long time.

**Table 1:** Laboratory values at admission.

**Test**	**Value**	**Value range**
Sodium	139 meq/l	135–145
Potassium	3.5 meq/l	3.5–4.9
Chloride	108 meq/l	96–110
CO_2_	28 mmol/l	20–30
BUN	18 mg/dl	6.0–23.0
Creatnine	0.7 mg/dl	0.6–1.4
Total protein	5.4 g/dl	6.0–8.0
Albumin	3.4 g/dl	3.6–5.0
Magnesium	1.6 meq/l	1.3–2.2
Phosphorus	3.3 mg/dl	2.5–4.5
Calcium	8.2 mg/dl	8.0–10.5
White blood count	12,400/mm^3^	4–12
Hemoglobin	11.6 g/dl	12.6–16.5
Platelet count	133 000/mm^3^	150–400
Uric acid	6.8 mg/dL	3.4–7.0

**Table 2:** Synovial fluid analysis.

**Test**	**Value**	**Normal**
Clarity	Translucent	Transparent
Color	Yellow	Clear
WBC (per mm^3^)	2100	< 200
PMNs (%)	62	< 25
Gram stain	No organisms	No organisms
Culture	Negative	Negative
Total protein (g/dL)	2	3.1
LDH (IU/L)	440	105 – 333
Glucose (mg/dL)	42	70–110
Crystal	Monosodium urate crystals	None

The patient responded partially to colchicine, but later had great relief with indomethacin. Colchicine was used at the dose of 0.6 mg every two hourly. He received total of six doses, but it was stopped because he developed severe nausea and vomiting. He admitted that his pain was reduced to 4/10 in intensity from 9/10 before treatment, but swelling was persistent. We initiated indomethacin at 50 mg every eight hourly, and his pain and swelling was relieved to great extent in 48 hours.

## Discussion

Gout is a metabolic disease that can manifest as acute or chronic arthritis, and deposition of urate crystals in connective tissue and kidneys. All patients have hyperuricemia at some point of their disease. But, either normal or low serum uric acid levels can occur at the time of acute attack; and asymptomatic hyperuricemic individuals may never experience a clinical event resulting from urate crystal deposition [1-4]. Low to normal uric acid concentration can be due to excessive excretion of uric acid, crystal formation, or systemic inflammatory state [6,7]; however, exact mechanism is still not completely understood. A diagnosis of gout is most accurate when supported by visualization of uric acid crystals in a sample of joint or bursal fluid, or demonstrated histologically in excised tissue. Synovial fluid analysis of our patient was consistent with inflammatory arthritis. Mild leucocytosis in this patient was due to systemic inflammatory response.

Visible or palpable tophi, as this patient exhibited, are usually noted only among those patients who are hyperuricemic and have had repeated attacks of acute gout, often over many years. However, presentation of tophaceous deposits in the absence of gouty arthritis is also reported [5,8]. Pain and inflammation are manifested when uric acid crystals activate the humoral and cellular inflammatory processes [9].

During an acute illness, if systemic inflammatory state prevails, such as in an acute infection, cytokines and chemokines triggers inflammation and cause arthritis in the presence of urate crystals [10,11]. Phagocytosis of these crystals by macrophages in the synovial lining cells precedes influx of neutrophils in the joint [9-11]. This process releases various mediators of inflammation locally [12,13].

Hyperuricemia is often present in patients with tophaceous gout, and they can benefit from uric acid lowering therapy early during the course [14,15]. In our patient, serum uric acid and 24-hour urine uric acid level was within normal limits when measured in the hospital before his discharge from the hospital. It was decided to follow him up in the clinic in two weeks, and measure these values again during 'interval gout' before deciding to start him on any particular medication to prevent further attacks of acute arthritis.

Our patient presented with tophi as an initial presentation of gout, which is very rare, but has been reported [5,8]. Investigational studies due to acute elbow joint pain deciphered the underlying mystery of chronic swelling. Systemic inflammatory response secondary to diverticulitis exposed the joints to the effects of urate.

First-line treatments for an acute flare are either oral colchicine and/or non-steroidal anti-inflammatory agents. Systemic or intra-articular corticosteroids can also be used, and are equally effective, but with more side effects [16,17]. Interleukin-1 inhibitors are still under investigation, and are not approved for an acute attack of gout [18].

## Conclusion

Gout usually flares up during an acute illness, and should always be considered while evaluating acute mono articular arthritis in hospitalized patients. Gout can present with tophi as an initial manifestation of the disease process.

## Competing interests

The authors declare that they have no competing interests.

## Authors' contributions

All authors contributed equally in collecting patient data, chart review, and editing medical images. All authors read and approved the final manuscript.

## Consent

Written informed consent was obtained from the patient for publication of this case report and accompanying images in Journal of Medical Case Reports. A copy of the written consent is available for review by the Editor-in-Chief of this journal.
